# The Association between the Plasma Phospholipid Profile and Insulin Resistance: A Population-Based Cross-Section Study from the China Adult Chronic Disease and Nutrition Surveillance

**DOI:** 10.3390/nu16081205

**Published:** 2024-04-18

**Authors:** Shao-Jie Pang, Ting-Ting Liu, Jian-Cun Pan, Qing-Qing Man, Shuang Song, Jian Zhang

**Affiliations:** 1 National Institute for Nutrition and Health, Chinese Center for Disease Control and Prevention, No. 29 of Nanwei Road, Beijing 100050, China; pangshaojie@feihe.com (S.-J.P.); liutt@ninh.chinacdc.cn (T.-T.L.); manqq@ninh.chinacdc.cn (Q.-Q.M.); 2 Key Laboratory of Public Nutrition and Health, National Health Commission of the People’s Republic of China, Beijing 100050, China; 3 Heilongjiang Feihe Dairy Co., Ltd., C-16, 10A Jiuxianqiao Rd., Beijing 100015, China; panjiancun@feihe.com

**Keywords:** phospholipids, insulin resistance, biomarker, HOMA-IR

## Abstract

The dysfunction of phospholipid metabolism enzymes and the change in membrane phospholipid composition are associated with insulin resistance, indicating that phospholipids play an important role in the regulation of insulin sensitivity. The reflection of phospholipid changes in blood might provide clues for both mechanism understanding and intervention. Using a targeted phospholipidomic approach, 199 phospholipid molecular species were identified and quantified in the plasma of 1053 middle-aged participants from a national investigation. The associations of the phospholipid matrix, clusters, and molecular species with insulin resistance were investigated. A significant association was confirmed between the phospholipid matrix and the homeostatic-model assessment of insulin resistance (HOMA-IR) by a distance-based linear model. Furthermore, three clustered phospholipid modules and 32 phospholipid molecular species were associated with HOMA-IR with the strict control of demographic and lifestyle parameters, family history of diabetes, BMI, WC, and blood lipid parameters. The overall decline in lysophosphatidylcholines (LPCs), the decrease in saturated lysophosphatidylethanolamines (LPEs), the decrease in polyunsaturated/plasmenyl phosphatidylcholines (PCs), and the increase in polyunsaturated phatidylethanolamines (PEs) were the prominent characters of plasma phospholipid perturbation associated with insulin resistance. This suggested that PC- and PE-related metabolic pathways were widely involved in the process of insulin resistance, especially the disorder of LPC acylation to diacyl-PC.

## 1. Introduction

Insulin resistance is a major contributor to the pathogenesis of type 2 diabetes (T2D), due to its effect on glucose disposal in muscle/fat and hepatic glucose output. And the hyperinsulinemia state of insulin resistance plays a key role in associated metabolic abnormalities, such as dyslipidemia and hypertension [1]. Thus, understanding the mechanisms of insulin resistance is essential for the development of preventive and therapeutic strategies for T2D and related diseases.

Previous studies have shown that the activity of insulin receptors, as their affinity to insulin, is dependent on the integrity and fluidity of the cell membrane [2,3,4]. As the major components of membranes, phospholipids could directly determine the biophysical properties of membranes, thereby affecting the function of membranes and membrane-bound proteins [5]. Epidemiologic studies have found associations between the fatty acyl saturation of phospholipids on cell membrane and insulin resistance [6,7,8]. And experimental studies at the animal-level have found several clues linking phospholipid metabolism to insulin resistance through the knockout of phospholipid synthesis enzymes, like phosphatidylethanolamine N-methyltransferase (PEMT) and lysophosphatidylcholine acyltransferase 3 (LPCAT3) [9,10,11,12]. Meanwhile, a growing number of plasma phospholipid species has been associated with related diseases of insulin resistance, like inflammation and mitochondrial dysfunction [13]. Also, a phosphatidylcholine (PC) molecular species was identified as an important endogenous ligand for peroxisome proliferator-activated receptor α (PPARα) in the liver, and the infusion of this phospholipid could decrease hepatic steatosis [14]. All these results have fueled speculation that phospholipids play an important role in the regulation of insulin sensitivity. The reflection of phospholipid changes in blood might not only help us to understand the mechanism of insulin resistance, but also provide clues for intervention.

However, the exact perturbations of the full-scale phospholipidome in vivo related to insulin resistance are still poorly understood. The main reason is that phospholipids are a complicated lipid family containing hundreds of phospholipid molecular species in bio-samples, and the precise identification and quantification of the phospholipidome, including classes, sub-classes, and individual molecular species, are always challenging. Limited by phospholipid-characterization techniques, most publications only focused on the association of insulin resistance and major classes of phospholipids, like PC and PE, without going into detailed molecular species and minor classes of phospholipids [15]. And the conclusions obtained by the few studies on the circulating phospholipidome are inconsistent [16,17]. The divergence of these conclusions might be due to the small sample size (*n* < 60) on the one hand, and the cross-interference of other metabolic processes on the other.

In this study, using liquid chromatography (LC) coupled with a high-resolution and a high-sensitivity mass spectrometry (MS), a total of 199 phospholipid molecular species were identified and quantified in the plasma of 1053 middle-aged participants. The associations between the phospholipidomic matrix, the phospholipid clusters, and the phospholipid molecular species with insulin resistance were comprehensively investigated. Other metabolic factors that may interfere with plasma phospholipid concentration and insulin resistance were considered and controlled stepwise to find a direct link between plasma phospholipids and insulin-resistance processes.

## 2. Materials and Methods

### 2.1. Study Population

The present study is based on the China Adult Chronic Disease and Nutrition Surveillance (2015), which is a nationwide cross-section survey focused on participants over 18 years old and pregnant women to assess the nutrition and health status of the Chinese adult population. The design of this surveillance has been described in detail in previously published articles [18]. Briefly, 302 surveillance points were selected from 31 provincial-level administrative divisions (PLADs) on mainland China according to a stratified, multistage, and random-sampling design, and 175,152 adult participants were investigated with the collection of questionnaires, anthropometric measurements, dietary surveys, and biological samples for laboratory tests (including fasting venous blood and urine samples). In the present study, 1140 participants aged 45–60 were selected from the surveillance points located in metropolises (municipalities, cities specifically designated in the state plan, or provincial capitals with more than one million civilians) by a simple random-sampling method for further analysis of the relationship between the phospholipidome and glycometabolism. To avoid the interference of medical treatment on insulin metabolism, 58 participants diagnosed with diabetes before the survey were excluded. At the same time, 29 participants with hemolytic or missing plasma samples were excluded as well. Eventually, a total of 1053 participants were included as the research objects of this study. The study protocol was approved by the ethical committee of the Chinese Center for Disease Control and Prevention (approval number: 201519-B; approval date: 15 June 2015). All the data and biological samples were collected with informed consent forms signed by the participants.

### 2.2. Basic Data Collection

A standardized questionnaire including information related to demographics, lifestyles, physical activity, health status, medical history, and family disease history was used and completed by strictly trained physicians or public health workers in face-to-face interviews with each participant. The educational attainment of the participants was categorized into 3 groups according to the number of years of education (≤6, 7–9, and ≥10 years). The college-educated and high-school-educated participants were combined as one group, since very few people aged 45–60 in 2015 received a college education (<3%). Current smoking refers to smoking at least one cigarette in the past 30 days; current alcohol drinking refers to drinking alcoholic beverages more than once a month on average in the past 12 months. Regular physical activity was defined as participating in medium/high intensity physical exercises one or more times a week, and lasting at least 10 min each time. Family history of diabetes was defined as a parent or sibling having diabetes.

### 2.3. Anthropometric Measurements

All participants were invited to have a physical examination at the local community hospitals. Participants were required to fast overnight, and to avoid vigorous exercise, smoking, drinking, eating, or long exposure to cold/hot temperatures before the examination. Anthropometric measurements were performed by trained medical professionals using a standardized protocol. Body weight and height were measured with light clothes and no shoes, and accurate to 0.1 kg and 0.1 cm, respectively. Body mass index (BMI) was calculated as weight (kg)/height^2^ (m^2^). The waist circumference (WC) was measured on the midpoint between the lower edge of the lowest costal arch and the upper edge of the iliac crest, and accurate to 0.1 cm. All the measurements were repeated two times, and the mean value was used for analysis.

### 2.4. Laboratory Measurements

Peripheral venous blood samples were collected from all participants after 10–14 h of fasting overnight. Serum samples were collected using serum separator tubes (SSTs) containing a gel phase. The SSTs were temporarily stored in an ice bath after collection, and centrifuged within 30 min, so serum could be separated from cellular components, and glycolysis could be stopped. This method could effectively reduce the drop in blood glucose levels and ensure their accuracy [19,20]. Whole blood and plasma samples were collected by EDTA-K2 vacuum tubes. All the samples were centrifuged at 3000 rpm for 10 min within 1 h after sampling. Serum, plasma, and whole blood were all divided into 0.5 mL and packed in tubes for cryogenic storage. All the samples were transported in dry ice to the central laboratory of the National Institute for Nutrition and Health and stored at −80 °C before analysis. Fasting glucose (Glu), total cholesterol (TC), triglycerides (TG), low-density lipoprotein cholesterol (LDL-C), and high-density lipoprotein cholesterol (HDL-C) were measured in serum enzymatically on a Hitachi 7600 automated biochemical analyzer (Hitachi, Tokyo, Japan) with reagents purchased from Wako Pure Chemical Industries (Osaka, Japan). High-performance affinity chromatography (Premier Hb9210, Trinity Biotech, Bray, Ireland) was used to measure glycohemoglobin (HbA_1c_) in whole blood. Fasting insulin (Ins) was measured in plasma using a Roche E601 automated chemiluminescence analyzer (Roche, Switzerland) with reagents purchased from Roche Diagnostics GmbH (Rotkreuz, Switzerland). The homeostatic-model assessment of insulin resistance (HOMA-IR) was calculated as Glu (mmol/L) × Ins (μU/mL)/22.5. The homeostatic-model assessment of β-cell function (HOMA-β) was calculated as 20 × Ins (μU/mL)/(Glu (mmol/L) − 3.5).

### 2.5. Phospholipidomic Measurement

The phospholipid molecular species in plasma were identified using a liquid chromatography–electrospray ionization ion-trap time-of-flight mass-spectrometry and further quantified by a liquid chromatography–electrospray ionization tandem mass-spectrometry (as described in Text S1). These two methods were validated in previously published research [21,22]. Briefly, a quality-control (QC) sample was prepared by taking 10 μL each from 1053 plasma samples and mixing them together. Using a modified Folch method, the total lipid was extracted from the QC sample for the qualitative analysis of phospholipids on a Shimadzu LC-ESI-IT-TOF system (Shimadzu, Kyoto, Japan). The phospholipids were preliminarily identified by matching MS data to the LIPID MAPS Structure Database (LMSD), and detailed structures of individual molecular species were further confirmed using MS2 and MS3 data obtained in multiple collisions. Eventually, 199 phospholipid molecular species were identified, including 5 phosphatidylglycerols (PGs), 18 phosphatidylinositols (PIs), 53 phosphatidylethanolamines (PEs), 60 phosphatidylcholines (PCs), 11 lysophosphatidylethanolamines (LPEs), 22 lysophosphatidylcholines (LPCs), and 30 sphingomyelins (SMs). Then, the phospholipid profiles of 1053 individual plasma samples were quantified using a Shimadzu 8060 LC-ESI-MS/MS system (Shimadzu, Japan) with a chromatogram collected in the targeted MRM mode and concentration calculated using an internal standard method. All the plasma samples were analyzed in a random order. A QC sample and a blank sample were added in the sequence after every 25 samples to ensure repeatability and no significant carryover of lipids, respectively. Finally, a total of 199 phospholipids were quantified in 1053 plasma samples (as shown in Table S1).

### 2.6. Statistical Analysis

The phospholipid concentrations below the detection limit were imputed as half of the limit of detection (LOD) of the corresponding phospholipid standards, and all the phospholipid concentrations were log-transformed for further statistical analysis. SAS, version 9.4S (SAS Institute, Inc., Cary, NC, USA), was used to perform Spearman correlation coefficients (*r*_spearman_) among phospholipid molecular species. PRIMER-E, version 7.0 (Quest Research Limited, Auckland, New Zealand), was used to perform a distance-based linear model (DistLM) analysis and to identify the association of lipidemic/glycemic parameters with the phospholipidomic matrix. District of residence, gender, age, educational attainment, marital status, current smoking, current alcohol drinking, regular physical activity, and family history of diabetes were forced into the model as adjustments. Bray–Curtis distances were used for the calculation of the resemblance matrix, and the permutation was performed 9999 times. Weighted gene co-expression-network analysis (WGCNA) was applied to study phospholipid networks and define modules of highly interconnected phospholipids (R package WGCNA 1.63, Vienna, Austria). The network interconnectedness of phospholipids was measured by topological overlap measure, and network plotting was performed (Cytoscape 3.8.2, National Institute of General Medical Sciences, Bethesda, MD, USA). The associations between phospholipid modules and individual molecular species with HOMA-IR were evaluated by calculating partial Spearman’s correlation coefficients (rspearman) in 4 models with stepwise adjustments of district of residence, gender, age, educational attainment, marital status, current smoking, current alcohol drinking, regular physical activity, family history of diabetes, BMI, WC, TC, TG, LDL-C, and HDL-C (SAS 9.4, SAS Institute, Inc., Cary, NC, USA). Stratified analyses were further performed for different groups of gender, current smoking, current alcohol drinking, regular physical activity, BMI, and WC. All significance levels were corrected for multiple testing with the false discovery rate (FDR) method, and an adjusted *p*-value < 0.05 was considered statistically significant for all the analyses.

## 3. Results

### 3.1. Basic Characteristics of Subjects

As shown in Table 1, a total of 1053 participants with mean a age of 53.1 years were involved in the present study, of which 527 (50.1%) were male and 142 (13.5%) participants reported a family history of diabetes. The general characteristics of participants were summarized in Table 1, which provides the count and proportion for classified variables, mean values, and standard deviations for normally distributed variables (like BMI, WC, TC, LDL-C, and Glu), median values, and upper/lower quartile values for skewed distribution variables (like TG, HDL-C, Ins, HbA_1c_, HOMA-IR, and HOMA-β).

### 3.2. Phospholipidomic Characteristics of Subjects

A total of 199 phospholipid molecular species, including 60 PC, 53 PE, 30 SM, 18 PI, 5 PG, 22 LPC, and 11 LPE species, were identified in the plasma of the 1053 participants. The concentration distribution of phospholipid species is shown in Table S1, and the concentration distribution of each phospholipid class is listed in Table 2. The median value of total phospholipid concentration in plasma was 2007.2 mg/L. And PC was the most abundant and diverse phospholipid class.

### 3.3. Association of Plasma Phospholipid Profile with Lipidemic and Glycemic Parameters

The significance of associations between the plasma phospholipid matrix and lipidemic/glycemic parameters was investigated using DistLM analysis based on a permutation test. With controls of district of residence, gender, age, educational attainment, marital status, current smoking, current alcohol drinking, regular physical activity, and family history of diabetes, all the lipidemic parameters (including BMI, WC, TC, TG, LDL-C, and HDL-C) were significantly associated with the variation in the plasma phospholipid matrix (*p* < 0.01 adjusted with the FDR method). Among all the glycemic parameters, only Ins and HOMA-IR were significantly associated with the variation of phospholipid matrix as shown in Table 3, while Glu, HbA_1c_, and HOMA-β were not associated with the variation in the plasma phospholipid matrix.

### 3.4. Association of Phospholipid Clusters with Insulin Resistance

**Figure 1:**
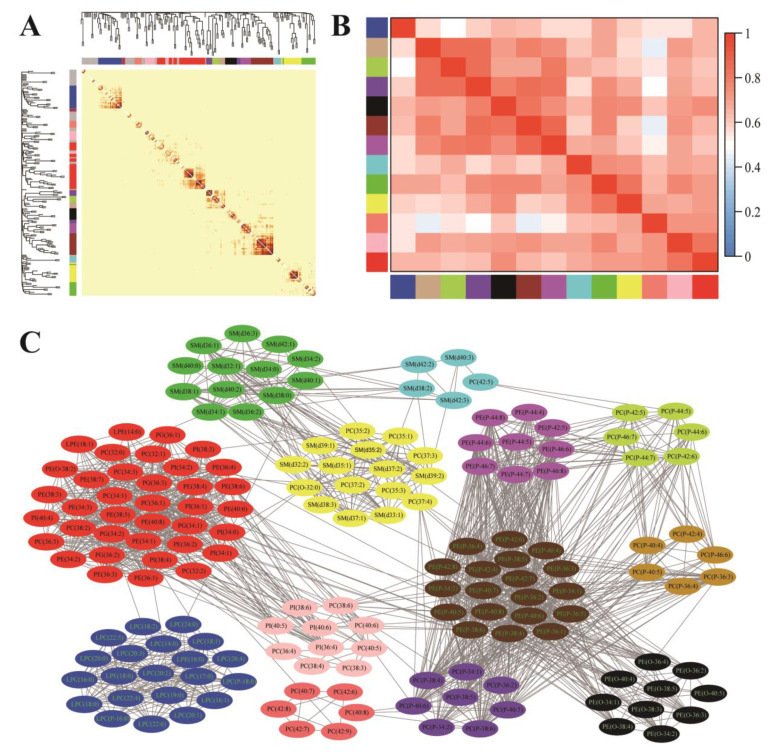
Weighted gene co-expression-network analysis (WGCNA) of plasma phospholipid molecular species. (**A**) Cluster dendrogram and correlation heatmap among 199 phospholipid molecular species (color bars represent phospholipid modules, and phospholipids that could not be clustered to any module were labeled gray). (**B**) Correlation heatmap among the 13 identified modules (color bars represent modules, and grid squares indicate Spearman’s correlation coefficients among module eigengenes. (**C**) The modules detected by topological overlap measure (blue: LPCs and LPEs; tan, green-yellow and purple: plasmenyl-PCs; black: plasmanyl-PEs; brown and magenta: plasmenyl-PEs; cyan and green: SMs; yellow: SMs and diacyl-PCs (most with odd-chain fatty acids); salmon: diacyl-PCs; pink: PIs and diacyl-PCs; red: PGs, PEs, PCs, PIs, and LPEs).

As demonstrated in Figure 1, the 199 plasma phospholipid molecular species were clustered into 13 modules via WGCNA analysis: module blue was composed of LPCs and LPE species; module tan, green-yellow, and purple were composed of plasmenyl-PC species (the fatty acid at *sn*-1 position is connected to glyceryl backbone with the vinyl ether bond); module black was composed of plasmanyl-PE species (the fatty acid at *sn*-1 position is connected to the glyceryl backbone with the ether bond); module brown and magenta were composed of plasmenyl-PE species; module cyan and green were mainly composed of SM species; module yellow was composed of SM and diacyl-PC species (most with odd-chain fatty acids); module salmon was composed of diacyl-PC species; module pink was composed of PI and diacyl-PC species; and module red was composed of PG, PE, PC, PI, and LPE species. The attribution of each phospholipid molecular species among these modules is listed in Table S1.

With the control of demographic and lifestyle parameters (district of residence, gender, age, educational attainment, marital status, current smoking, current alcohol drinking, and regular physical activity), seven modules (blue, tan, green-yellow, purple, magenta, cyan, and salmon) were negatively correlated with HOMA-IR, as shown in Table 4, while two modules (pink and red) were positively associated with HOMA-IR (*p* < 0.05 after multiple tests with the FDR method). The further adjustment of the family history of diabetes affected neither the significance nor direction of the correlation. But the associations between HOMA-IR and three modules (tan, black, and magenta) composed of ether phospholipids (plasmenyl/plasmanyl) were abolished when BMI and WC were added as covariates. And three modules (green-yellow, cyan, and red) were no longer associated with HOMA-IR with the further control of blood lipid parameters (TC, TG, LDL-C, and HDL-C). Finally, only three modules (blue, salmon, and pink) containing LPC, LPE, diacyl-PC, and PI species were still correlated with HOMA-IR; and module blue, containing LPC and LPE species, presented the highest correlation coefficient with HOMA-IR.

### 3.5. Association of Phospholipid Molecular Species with Insulin Resistance

The association between phospholipid molecular species and HOMA-IR was also investigated using the four models described in the last section. With the control of demographic and lifestyle parameters, 75 individual phospholipid molecular species (including 20 PC, 21 PE, 5 SM, 2 PI, 4 PG, 19 LPC, and 4 LPE species) were significantly correlated with HOMA-IR, as listed in Table 5. And the further adjustment of the family history of diabetes did not affect the significance and direction of the correlation. When BMI, WC, and blood lipid parameters were progressively adjusted as covariates, 12 PC, 19 PE, 1 PI, 1 LPC, 1 LPE, and all the SM and PG species were no longer associated with HOMA-IR. Thus, only 32 phospholipid molecular species (8 PC, 2 PE, 1 PI, 18 LPC, and 3 LPE species) were significantly associated with HOMA-IR, most of which were negatively associated with HOMA-IR except 2 PE and 1 PI species. Of note, lysophospholipid species, especially abundant LPC species, present a stronger correlation with insulin resistance compared with other phospholipid species.

**Figure 2:**
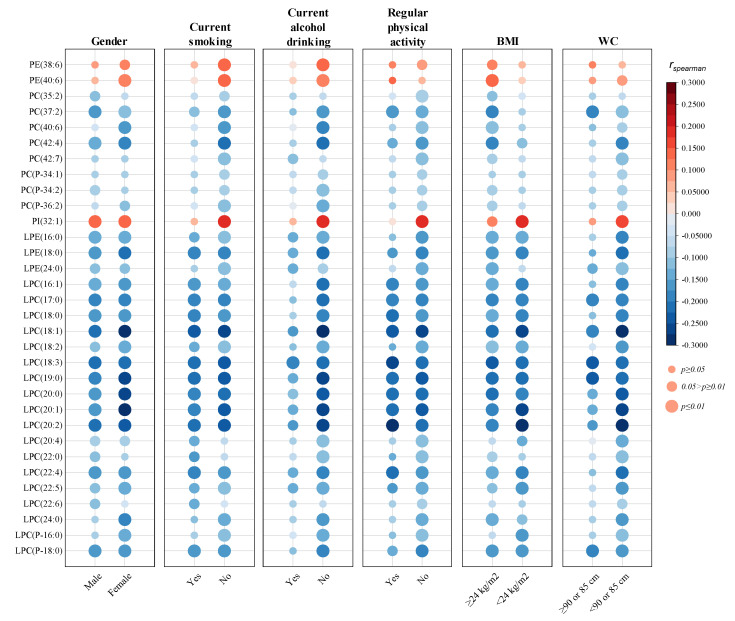
Stratified analyses of the associations between plasma phospholipids and HOMA-IR. District of residence, gender, age, educational attainment, marital status, current smoking, current alcohol drinking, regular physical activity, family history of diabetes, BMI, WC, TC, TG, LDL-C, and HDL-C were adjusted as covariates. *r_spearman_*: spearman correlation coefficient; *p* values were adjusted by multiple tests with FDR method.

Furthermore, the associations between HOMA-IR and the 32 phospholipid molecular species were stratified based on gender, current smoking, current alcohol drinking, regular physical activity, BMI, and WC. As demonstrated in Figure 2, no apparent differences were observed when analyses were stratified by gender. However, the associations of PC, PE, and PI species with HOMA-IR were mostly abolished or weakened among individuals with a habit of smoking, alcohol drinking, or regular physical activity, and individuals with a lower BMI (<24 kg/m^2^) or a larger WC (≥90 cm for males and ≥85 cm for females). In contrast, the associations between HOMA-IR and lysophospholipids were more stable in stratified analyses, only weakened among participants with an alcohol-drinking behavior or a larger WC.

## 4. Discussion

In the present study, we have shown that the changes in plasma phospholipid matrixes were significantly correlated with insulin level and insulin resistance rather than glucose level, glycosylation degree, and β-cell function, suggesting that there is a disruption in phospholipid metabolism presented along with insulin resistance.

The most prominent perturbation of the phospholipid matrix associated with insulin resistance was the decreased concentrations of LPCs. Almost all the LPC species, regardless of fatty acyl structures, were significantly reduced along with increased HOMO-IR under the control of covariates. These changes coincided with the association between circulating insulin and the expression of LPCAT3, an enzyme involved in the transacylation of LPC to synthesize PC (a part of Lands’ cycle), in skeletal muscle that was reported in animal-level experiment studies: the knockout of LPCAT3 mediated LPC accumulation in muscle and improved insulin sensitivity under a high-fat diet, while the overexpression of LPCAT3 in skeletal muscle exhibited decreased LPC in muscle cells and promoted glucose intolerance [11]. LPC metabolism was considered a key pathway that mediated the progression from obesity to insulin resistance, since a decrease in circulating LPC was also widely observed among overweight and obese individuals [23,24]. However, the present study found that the associations between most LPCs and insulin resistance were substantially attenuated among participants with larger WC, which is inconsistent with the view that obesity-induced LPC decrease would promote insulin resistance. Similarly, decreased serum LPC levels were observed in patients with alcoholic liver cirrhosis [25], and attenuated associations between most LPCs and insulin resistance were observed among individuals with alcohol drinking in the present study. Since obesity and alcohol consumption both affect lipid metabolism in the liver, and blood reflects the metabolism of multiple organs, we speculate that liver and skeletal muscle may cause relatively independent LPC perturbation, muscle-derived LPC perturbations could better predict insulin resistance, and liver-derived LPC perturbations are not necessarily associated with insulin resistance. In addition, three LPEs were found to be associated with insulin resistance in the present study. Unlike almost all the plasma LPCs decreased along with insulin resistance, these LPEs associated with HOMA-IR contained only saturated fatty acyl groups, indicating that LPEs might be involved in insulin resistance in a different way to LPCs, but the mechanism and significance of this difference is unclear.

The other notable perturbation of circulating phospholipids correlated with insulin resistance in the present study was the decreased concentrations of eight PC species (including five diacyl-PCs and three plasmenyl-PCs). It is interesting that the general reduction of LPC associated with insulin resistance did not lead to a decreased concentration for most PCs, indicating that there are other sources of PC in addition to LPC acylation. In fact, PC could be synthesized via three different pathways: the de novo synthesis Kennedy pathway, the Lands’ cycle, as mentioned above, and the PEMT pathway [26]. According to animal-level experiment studies, the knockout of PEMT, the critical enzyme for the conversion of PE to PC, resulted in a significant decrease in hepatic PC:PE ratio and insulin resistance [10,27,28], suggesting that the changes in PC-PE balance could affect the insulin sensitivity in vivo. And a deficiency of muscle CEPT1, the terminal enzyme for de novo synthesis of PC in the Kennedy pathway, resulted in a significant reduction of two diacyl-PEs and an significant increase in three diacyl-PCs containing docosahexaenoic acyl (DHA) or arachidonic acyl (AA) moieties in muscle endoplasmic reticulum, and improved insulin sensitivity in high-fat-diet mice [9,29]. In the present study, the circulating levels of two diacyl-PEs and three diacyl-PCs containing DHA or AA moieties, including PE(38:6), PE(40:6)), PC(40:6), PC(42:4), and PC(42:7), were also found to be related to insulin resistance. Although the mechanism under this association is not fully understood, an increased proportion of PEs and a decreased proportion of PCs (especially species with a polyunsaturated acyl moiety) in the bilayer membrane were related to a more hexagonal HII phase (a non-lamellar phase assembled via lipids) that could decrease Ca^2+^-ATPase activity, and, inactive of sarco/endoplasmic reticulum Ca^2+^-ATPase (SERCA), would cause the dysregulation of the endoplasmic reticulum Ca^2+^ homeostasis that is important for insulin-signal transduction [30,31]. Furthermore, the correlation between circulating plasmenyl-PCs and insulin resistance observed among the participants of the present study is very similar to the changes of plasmalogens among insulin-resistant mice induced by a high-fat diet [32]. However, our knowledge of the role of plasmalogens in insulin resistance is still limited to their antioxidant effects derived from the vinyl ether bond, and more research is needed to find the sites and specific pathways of other effects.

In addition to PC, PE, and their lyso-type, other classes of phospholipid in low abundance (like PG, PI, and SM) are also associated with inflammation and mitochondrial function [33,34,35,36], and therefore might participate in the progression of insulin resistance. However, the correlations between PGs/SMs and HOMA-IR in both clustered modules and single molecular species were weakened and no longer significant after the adjustment of BMI, WC, and blood lipidemic indexes in the present study. Only plasma PI(32:1) was positively associated with HOMA-IR. Although this is the first time that plasma PI was connected to insulin resistance, the expression of phosphatidylinositol kinases that are responsible for the conversion of PIs to phosphorylated derivatives (including PIP, PIP2, and PIP3) was reported to be positively correlated with insulin sensitivity [37,38]. We suspect that the increased PI concentration associated with insulin resistance might be a consequence of the obstruction of the phosphorylation pathway.

Furthermore, when BMI, WC, and blood lipid parameters were adjusted, all the plasmalogens containing long-chain polyunsaturated fatty acyl (LCPUFA) groups, SMs, and PGs were no longer associated with HOMA-IR. Although plasmalogen was previously reported to be abundant in brain and heart tissues, Lange et al. recently demonstrated that plasmalogen is also enriched in human white adipose tissue [39]. The content of LCPUFA-containing plasmalogens was found to be reduced in the adipose tissue of obese people and mice [40], and the supplement of alkylglycerol (precursor of plasmalogen containing LCPUFA) among overweight or obese subjects could increase plasmalogen levels and reduce TC, TG, and C-reactive protein levels in plasma [41]. Moreover, a phospholipidomics analysis has found that serum PGs were elevated in patients with low microbiota gene richness, and excess PGs may favor the maintenance of adiposity by modulating adipocyte lipid mobilization [42]. These pieces of evidence are consistent with the results of the present study, and indicate that the deficiency of LCPUFA-containing plasmalogens and the increase in PGs might be part of the causal chain between obesity/dyslipidemia and insulin resistance. As for SM, previous studies on human subjects reported that several SM species with saturated or monounsaturated acyl chains were positively correlated with obesity, blood lipid parameters, and insulin resistance [43]. Although this suggests that SM may participate in obesity/dyslipidemia-induced insulin resistance, the correlation direction between SM species and insulin resistance observed in the present study is opposite to that reported in the previous studies. The reason for this phenomenon is currently unclear, and further verification the with strict control of sampling and the use of a standardized analytical procedure is required.

A strength of the present study is the accurate quantitative and qualitative measurements of nearly 200 phospholipid molecular species in tandem. In addition, the subjects of this study come from a national survey (the China Adult Chronic Disease and Nutrition Surveillance) with a nationally representative sampling design, standardized collection of data, strict quality-control scheme, and few missing data. The sample size of this study exceeds 1000 cases, which is the largest scale population-based investigation discussing the relationship between phospholipids and insulin resistance to our knowledge. The cross-sectional nature of the present study is a limitation that does not allow us to determine whether perturbations in phospholipid metabolism are causal or a consequence of insulin resistance. This limitation could be elucidated in prospective cohort studies with repeated measurements of circulating phospholipids in the population at risk of developing insulin resistance. Furthermore, even with FDR-adjusted *p*-values and the careful control of interfering factors, some of the associations found in the present study might still be the result of chance. To further confirm the phospholipid molecules that are actually involved in insulin resistance, animal experiments involving interventions of a specific metabolic pathway through gene knockout or dietary supplementation are needed.

## 5. Conclusions

The present study provides evidence that the plasma phospholipid profile changes along with insulin resistance. The overall decline in LPCs, the decrease in saturated LPEs, the decrease in polyunsaturated/plasmenyl PCs, and the increase in polyunsaturated PEs were the most prominent characteristics of plasma phospholipid perturbation associated with insulin resistance. This suggested that PC- and PE-related metabolic pathways were involved in the process of insulin resistance, especially the disorder of LPC acylation to diacyl-PC. Further intervention studies, including more large-scale cohort studies or randomized clinical trials, are warranted to assess these results and seek interventions targeting the improvement of insulin resistance and T2D.

## Figures and Tables

**Table 1:** Basic characteristics of participants (*n* = 1053).

Characteristics	Mean (SD) or Median (Q1, Q3) or *n* (%)
Gender (male)	527 (50.1%)
Age (years)	53.1 (4.6)
Educational attainment,	
≤6 years	527 (50.1%)
7–9 years	370 (35.1%)
≥10 years	156 (14.8%)
Marital status, married or living with a partner	1003 (95.3%)
Current smoking	308 (29.3%)
Current alcohol drinking	305 (29.0%)
Regular physical activity	309 (29.3%)
Family history of diabetes	142 (13.5%)
BMI (kg/m^2^)	24.8 (3.7)
WC (cm)	84.5 (9.6)
TC (mmol/L)	4.9 (0.9)
TG (mmol/L)	1.3 (0.9–1.9)
LDL-C (mmol/L)	3.1 (0.8)
HDL-C (mmol/L)	1.2 (1.0, 1.5)
Glu (mmol/L)	5.6 (1.5)
Ins (µIU/mL)	7.8 (5.3, 11.2)
HbA_1c_ (%)	5.0 (4.6, 5.5)
HOMA-IR	1.8 (1.2, 3.0)
HOMA-β	91.2 (59.7, 138.3)

SD: standard deviation; Q1: lower quartile; Q3: upper quartile; BMI: body mass index; WC: waist circumference; TC: total cholesterol; TG: triglycerides; LDL-C: low-density lipoprotein cholesterol; HDL-C: high-density lipoprotein cholesterol; Glu: fasting glucose; Ins: fasting insulin; HbA_1c_: glycohemoglobin; HOMA-IR: homeostasis-model assessment for insulin resistance; and HOMA-β: homeostatic-model assessment of β-cell function.

**Table 2:** Plasma phospholipid composition of participants (*n* = 1053).

Classes	Molecular Species (*n*)	Concentration (mg/L)		
		Median	Q1	Q3
PC	60	1412.4	1187.6	1639.2
PE	53	36.6	27.0	48.4
SM	30	346.5	295.9	399.5
PI	18	33.7	25.7	41.6
PG	5	0.5	0.4	0.8
LPC	22	151.1	123.7	187.4
LPE	11	3.4	2.5	4.3
Total	199	2007.2	1753.6	2280.7

Q1: lower quartile; Q3: upper quartile; PC: phosphatidylcholine; PE: phosphatidylethanolamine; SM: sphingomyelin; PI: phosphatidylinositol; PG: phosphatidylglycerol; LPC: lysophosphatidylcholine; and LPE: lysophosphatidylethanolamine.

**Table 3:** Distance-based linear model (DistLM) results between phospholipidomic matrix and lipidemic/glycemic parameters.

	*R_adj_* ^2^	*SS_trace_*	*Pseudo-F* Value	*p* Value
BMI	0.118	241.19	10.18	<0.001 *
WC	0.120	92.27	3.91	0.007 *
TC	0.223	2892.70	138.61	<0.001 *
TG	0.252	830.51	41.34	<0.001 *
LDL-C	0.266	402.66	20.42	<0.001 *
HDL-C	0.271	175.57	8.97	<0.001 *
Glu	0.272	37.09	1.90	0.065
Ins	0.274	73.57	3.77	0.003 *
HbA_1c_	0.275	33.19	1.70	0.106
HOMA-IR	0.276	67.20	3.46	0.002 *
HOMA-β	0.276	15.07	0.78	0.531

District of residence, gender, age, educational attainment, marital status, current smoking, current alcohol drinking, regular physical activity, and family history of diabetes were forced into the model as controls. *R_adj_*^2^: adjusted determination coefficient; *SS_trace_*: sum of squares of deviation from mean; *: *p* values that remained significant after multiple testing with FDR method.

**Table 4:** Spearman correlation coefficients between WGCNA module eigengenes and HOMA-IR.

Module	Median (Q1, Q3)	*r_spearman_* with HOMA-IR			
		Model 1	Model 2	Model 3	Model 4
Module blue	0.21 (0.12,0.31)	−0.202 *	−0.201 *	−0.145 *	−0.200 *
Module tan	0.25 (0.09,0.39)	−0.115 *	−0.113 *	−0.019	0.010
Module green-yellow	0.22 (0.09,0.34)	−0.148 *	−0.146 *	−0.065 *	−0.039
Module purple	0.28 (0.13,0.49)	−0.142 *	−0.140 *	−0.054	−0.010
Module black	0.37 (0.25,0.47)	−0.043	−0.041	0.028	0.019
Module brown	0.31 (0.15,0.50)	−0.047	−0.046	0.038	0.026
Module magenta	0.26 (0.11,0.46)	−0.165 *	−0.164 *	−0.051	−0.010
Module cyan	0.23 (0.14,0.35)	−0.130 *	−0.125 *	−0.080 *	−0.068
Module green	0.38 (0.26,0.47)	−0.020	−0.018	0.008	0.034
Module yellow	0.27 (0.18,0.43)	−0.052	−0.050	−0.055	−0.051
Module salmon	0.23 (0.06,0.34)	−0.072 *	−0.070 *	−0.066 *	−0.094 *
Module pink	0.37 (0.24,0.46)	0.093 *	0.094 *	0.147 *	0.081 *
Module red	0.33 (0.25,0.51)	0.125 *	0.125 *	0.132 *	−0.024

Model 1 controlled district of residence, gender, age, educational attainment, marital status, current smoking, current alcohol drinking, and regular physical activity; Model 2 further controlled family history of diabetes; Model 3 further controlled BMI and WC; Model 4 further controlled TC, TG, LDL-C, and HDL-C. Q1: lower quartile; Q3: upper quartile; rspearman: spearman correlation coefficient; *: correlation coefficients with *p* values that remained significant (<0.05) after multiple tests with FDR method.

**Table 5:** Spearman correlation coefficients between phospholipid molecular species and HOMA-IR.

No.	Name	*r_spearman_* with HOMA-IR					No.	Name	*r_spearman_* with HOMA-IR			
		Model 1	Model 2	Model 3	Model 4				Model 1	Model 2	Model 3	Model 4
1	PC(30:0)	0.13 *	0.13 *	0.06	−0.01		42	SM(d34:0)	−0.16 *	−0.16 *	−0.08 *	−0.05
2	PC(32:1)	0.14 *	0.15 *	0.12 *	0.03		43	SM(d34:1)	−0.16 *	−0.16 *	−0.06	<0.01
3	PC(32:2)	0.20 *	0.20 *	0.14 *	0.06		44	SM(d38:3)	−0.10 *	−0.10 *	−0.08 *	−0.07
4	PC(35:2)	−0.10 *	−0.10 *	−0.08 *	−0.08 *		45	SM(d42:2)	−0.13 *	−0.12 *	−0.06	−0.05
5	PC(36:5)	0.13 *	0.13 *	0.11 *	0.07		46	SM(d42:3)	−0.15 *	−0.15 *	−0.09 *	−0.07
6	PC(37:2)	−0.15 *	−0.15 *	−0.12 *	−0.14 *		47	PI(32:1)	0.23 *	0.23 *	0.19 *	0.14 *
7	PC(40:6)	−0.12 *	−0.12 *	−0.16 *	−0.10 *		48	PI(36:4)	0.10 *	0.10 *	0.11 *	0.02
8	PC(42:4)	−0.14 *	−0.14 *	−0.12 *	−0.16 *		49	PG(34:1)	0.13 *	0.13 *	0.15 *	−0.07
9	PC(42:7)	−0.10 *	−0.10 *	−0.08 *	−0.09 *		50	PG(34:2)	0.24 *	0.23 *	0.22 *	0.06
10	PC(P-34:1)	−0.24 *	−0.24 *	−0.15 *	−0.08 *		51	PG(36:1)	0.30 *	0.30 *	0.24 *	0.03
11	PC(P-34:2)	−0.19 *	−0.19 *	−0.14 *	−0.08 *		52	PG(36:2)	0.27 *	0.27 *	0.23 *	0.01
12	PC(P-36:2)	−0.22 *	−0.22 *	−0.14 *	−0.09 *		53	LPC(14:0)	0.13 *	0.13 *	0.07	−0.02
13	PC(P-40:7)	−0.11 *	−0.10 *	<0.01	0.04		54	LPC(16:1)	−0.10 *	−0.10 *	−0.10 *	−0.16 *
14	PC(P-42:4)	−0.14 *	−0.14 *	−0.05	−0.02		55	LPC(17:0)	−0.17 *	−0.17 *	−0.16 *	−0.19 *
15	PC(P-42:5)	−0.14 *	−0.13 *	−0.08 *	−0.06		56	LPC(18:0)	−0.13 *	−0.13 *	−0.11 *	−0.16 *
16	PC(P-44:5)	−0.14 *	−0.14 *	−0.09 *	−0.07		57	LPC(18:1)	−0.29 *	−0.28 *	−0.21 *	−0.24 *
17	PC(P-44:6)	−0.12 *	−0.12 *	−0.06	−0.03		58	LPC(18:2)	−0.24 *	−0.24 *	−0.14 *	−0.13 *
18	PC(P-44:7)	−0.14 *	−0.14 *	−0.05	−0.03		59	LPC(18:3)	−0.19 *	−0.19 *	−0.16 *	−0.21 *
19	PC(P-46:6)	−0.15 *	−0.15 *	−0.08 *	−0.05		60	LPC(19:0)	−0.25 *	−0.25 *	−0.19 *	−0.21 *
20	PC(P-46:7)	−0.15 *	−0.15 *	−0.05	−0.02		61	LPC(20:0)	−0.26 *	−0.26 *	−0.18 *	−0.20 *
21	PE(34:1)	0.20 *	0.20 *	0.17 *	0.01		62	LPC(20:1)	−0.27 *	−0.27 *	−0.20 *	−0.22 *
22	PE(34:2)	0.21 *	0.21 *	0.18 *	0.03		63	LPC(20:2)	−0.27 *	−0.27 *	−0.21 *	−0.24 *
23	PE(34:3)	0.12 *	0.12 *	0.08 *	−0.06		64	LPC(20:4)	−0.15 *	−0.15 *	−0.10 *	−0.10 *
24	PE(36:1)	0.20 *	0.20 *	0.17 *	0.02		65	LPC(22:0)	−0.13 *	−0.12 *	−0.10 *	−0.10 *
25	PE(36:2)	0.19 *	0.19 *	0.17 *	0.01		66	LPC(22:4)	−0.18 *	−0.18 *	−0.14 *	−0.18 *
26	PE(36:4)	0.15 *	0.15 *	0.16 *	0.04		67	LPC(22:5)	−0.16 *	−0.16 *	−0.10 *	−0.14 *
27	PE(38:3)	0.13 *	0.13 *	0.15 *	0.02		68	LPC(22:6)	−0.14 *	−0.14 *	−0.08 *	−0.08 *
28	PE(38:4)	0.14 *	0.14 *	0.15 *	0.02		69	LPC(24:0)	−0.22 *	−0.22 *	−0.12 *	−0.13 *
29	PE(38:6)	0.16 *	0.16 *	0.18 *	0.09 *		70	LPC(P-16:0)	−0.17 *	−0.17 *	−0.12 *	−0.10 *
30	PE(40:6)	0.24 *	0.24 *	0.23 *	0.11 *		71	LPC(P-18:0)	−0.14 *	−0.14 *	−0.13 *	−0.17 *
31	PE(P-36:1)	−0.11 *	−0.11 *	−0.05	0.01		72	LPE(14:0)	0.14 *	0.15 *	0.12 *	0.03
32	PE(P-36:2)	−0.10 *	−0.10 *	−0.02	0.04		73	LPE(16:0)	−0.10 *	−0.10 *	−0.08 *	−0.14 *
33	PE(P-42:5)	−0.11 *	−0.10 *	−0.02	0.02		74	LPE(18:0)	−0.13 *	−0.13 *	−0.08 *	−0.19 *
34	PE(P-44:4)	−0.11 *	−0.12 *	−0.03	−0.01		75	LPE(24:0)	−0.16 *	−0.16 *	−0.09 *	−0.10 *
35	PE(P-44:5)	−0.16 *	−0.15 *	−0.07 *	−0.05							
36	PE(P-44:6)	−0.14 *	−0.14 *	−0.04	0.01							
37	PE(P-44:7)	−0.15 *	−0.15 *	−0.04	<0.01							
38	PE(P-44:8)	−0.12 *	−0.12 *	−0.02	0.02							
39	PE(P-46:6)	−0.18 *	−0.18 *	−0.07	−0.03							
40	PE(P-46:7)	−0.18 *	−0.17 *	−0.07	−0.03							
41	PE(P-46:8)	−0.17 *	−0.17 *	−0.06	−0.01							

Model 1 controlled district of residence, gender, age, educational attainment, marital status, current smoking, current alcohol drinking, and regular physical activity; Model 2 further controlled family history of diabetes; Model 3 further controlled BMI and WC; Model 4 further controlled TC, TG, LDL-C, and HDL-C. rspearman: spearman correlation coefficient; *: correlation coefficients with *p* values that remained significant (<0.05) after multiple tests with FDR method.

## Data Availability

The original data are not allowed to be disclosed according to the National Institute for Nutrition and Health, Chinese Center for Disease Control and Prevention.

## Supplementary Materials

The following supporting information can be downloaded at https://www.mdpi.com/article/10.3390/nu16081205/s1, Text S1. Phospholipidomic analysis method; Table S1. Quantitative ions, concentration distribution and module information (WGCNA model) of the identified phospholipid molecular species.
