# Supplementary material for: The Association between the Plasma Phospholipid Profile and Insulin Resistance: A Population-Based Cross-Section Study from the China Adult Chronic Disease and Nutrition Surveillance
Source: Nutrients. 2024 Apr 18;16(8):1205. doi: 10.3390/nu16081205 (PMC11054597; doi:10.3390/nu16081205)
Supplement: Supplementary file 1 [file nutrients-16-01205-s001.zip › nutrients-2962186-supplementary.pdf]

Supplemental Material

Text S1. Phospholipidomic analysis method

Chemicals

PL standards and deuterium labeled internal PL standards (as listed below) were purchased from Avanti Polar Lipids (USA). Other chemicals, including chloroform, methanol, acetonitrile, ammonium formate and formic acid were purchased from Sigma Aldrich (USA) and of HPLC or MS grade. Ultra-pure water was supplied by a Milli-Q system (Millipore, USA).

List of phospholipid standards and deuterium labeled internal standards

	Exact Mass
PE(14:0/14:0)	635.453
PE(17:0/17:0)	719.547
PE(18:0/18:0)	747.578
PE(18:1/18:1)	743.547
PE(18:2/18:2)	739.515
PC(14:0/14:0)	677.500
PC(17:0/17:0)	761.593
PC(18:0/18:0)	789.625
PC(18:1/18:1)	785.593
PC(18:2/18:2)	781.562
PC(20:0/20:0)	845.687
PC(22:0/22:0)	
LPE(14:0)	425.254
LPE(16:0)	453.286
LPE(18:0)	481.317
LPE(18:1)	479.300
SM(d18:1/12:0)	646.505
SM(d18:1/16:0)	702.568
SM(d18:1/18:0)	730.599
SM(d18:1/18:1)	728.583
LPC(13:0)	453.286
LPC(17:0)	509.348
LPC(17:1)	507.332
LPC(19:0)	537.379
PG(14:0/14:0)	688.429
PG(16:0/16:0)	744.492
PG(18:0/18:0)	800.554
PG(18:1/18:1)	796.523
PI(16:0/16:0)	827.552
PI(18:0/18:0)	883.615
PI(18:1/18:1)	879.584
PC(15:0/18:1) (d7)	752.606
LPC(18:1) (d7)	528.392
PE(15:0/18:1) (d7)	710.559

LPE(18:1) (d7)	486.345
PG(15:0/18:1) (d7)	741.554
PI(15:0/18:1) (d7)	829.570
PS(15:0/18:1) (d7)	754.549
SM(d18:1/18:1) (d9)	737.640

Extraction of total phospholipid from human plasma

All the plasma samples were thawed at 37 ºC. Total PL was extracted from human plasma according to a modified Folch method. Briefly, 100 µL plasma, 10 µL deuterium labeled internal standards solution and 1.0 mL chloroform/methanol (2:1, v/v) were subjected to vortex mixing for 30 min. Then, 0.2 mL NaCl solution (0.9%) was added to the mixture. The resultant mixture was mixed for 30 min and centrifuged at 10,000 rpm for 10 min. The chloroform layer was transferred into a new tube and dried under a gentle stream of nitrogen. All the samples were redissolved in 100 µL of chloroform/methanol (2:1, v/v) before analyses.

Qualitative analysis of phospholipid molecular species in plasma

PL molecular species was identified according to a HILIC-ESI-IT-TOF-MS method described in our previous publication[1]. A mixed human plasma sample was prepared by taken 10 µL each from 1053 plasma samples and mixed together. This sample was used for qualitative analysis of phospholipid molecular species. Total PL was extracted from 500 µL mixed plasma and redissolved in 50 µL of chloroform/methanol (2:1, v/v). This concentrated sample was injected into HILIC-ESI-IT-TOF-MS system. Scan data of MS, MS^2 and MS^3 were collected under ESI^- mode. The MS data were collected in the range of 400-1000 m/z with 30 ms of ion accumulation in ion trap; the MS^2 data were collected in the range of 100-800 m/z with 50 ms of ion accumulation and 100% of collision energy; the MS^3 data were collected in the range of 100-700 m/z with 50 ms of ion accumulation and 100% of collision energy. All the data were collected at a loop time of 0.82 s. The PLs were preliminarily identified by comparing m/z values with calculated exact masses using LIPID MAPS Structure Database (LMSD). Detailed structures of individual molecular species were further confirmed by MS^2 and MS^3 spectra based on the fragmentation laws of PLs that described in our previous publication [1].

Quantitative analysis of phospholipid molecular species in human plasma

The identified PL molecular species was quantified according to a HILIC-ESI-MS/MS method. HPLC system (Shimadzu, Japan) equipped with binary pump (LC-20AD XR), online degasser (FCV-11AL), autosampler (SIL-30AC), and thermostatic column oven (CTO-20AC) was coupled online to a quadrupole mass spectrometer (LC-MS 8060, Shimadzu Japan) equipped with an Electrospray Ionization (ESI) source for plasma PL quantification.

Chromatography The extracted PLs were separated on a CORTECS HILIC Column (1.6 µm, 2.1 mm X 150

mm, Waters, USA). The column temperature was set at 40 ºC. Eluent A and B were water and acetonitrile/water (95/5), respectively. Both eluents contained 0.1% formic acid and 10 mM ammonium formate. The flow rate of the eluent was 0.4 mL/min. The elution gradient was as follows: 0 min, 99.5% B; 21 min, 91% B; 21.1 min, 80% B; 26 min, 80% B; 26.1 min, 99.5 % B; 32min, 99.5% B. No significant carryover of lipids was observed in blank injection (10 μL methanol) after every 25 samples.

Mass Spectrometry The instrument parameters were set as follows: nebulizer gas flow: 3 L/min; heat gas flow: 12 L/min; desolvation gas flow: 8 L/min; interface temperature: 250 ºC; DL temperature: 180 ºC; heat block temperature: 250 ºC; detector voltage: 1.85 kV. The ESI source operated in positive mode for PE, PC, LPE, LPC, and SM, and in negative mode for PI and PG. The MS/MS data were collected in targeted MRM mode for all the PL molecular species identified in HILIC-ESI-IT-TOF-MS system. The m/z values of precursor and product ions were calculated biased on the fragmentation laws of PLs in quadrupole system as described in our previous publication [2].

Quantification Individual molecular species of PL were quantified using internal standard method. Calibration curves with $1/x^2$ as weighting factor were constructed using PL standards in a series of concentrations spiked with deuterium labeled internal standards in constant concentration (as shown in Table S4 of supplementary material). The plasma PLs were quantified using calibration curve of the PL standard from same class and with most similar acyl chain length and unsaturation degree (as shown in Table S3 of supplementary material). A QC sample was added in the sequence after every 25 samples for quality control.

Reference

1. Song S, Cheong LZ, Man QQ, Pang SJ, Li YQ, et al. (2018) Characterization of potential plasma biomarkers related to cognitive impairment by untargeted profiling of phospholipids using the HILIC-ESI-IT-TOF-MS system. Anal Bioanal Chem 410: 2937-2948.
2. Song S, Liu TT, Liang X, Liu ZY, Yishake D, et al. (2021) Profiling of phospholipid molecular species in human breast milk of Chinese mothers and comprehensive analysis of phospholipidomic characteristics at different lactation stages. Food Chem 348: 129091.

Table S1. Quantitative ions, concentration distribution and module information (WGCNA model) of the identified phospholipid molecular species

No.	Molecular species	Precusor ion (m/z)	Product ion (m/z)	Concentration (μg/L)			WGCNA module
				Median	Q1	Q3	
1	PG(34:1)	747.5	281.3	228.3	322.2	455.5	red
2	PG(34:2)	745.5	279.3	78.8	119.1	172.9	red
3	PG(36:1)	775.5	281.3	23.4	35.2	51.6	red
4	PG(36:2)	773.5	279.3<br>281.3<br>327.3	32.3	44.3	62.6	red
5	PG(36:3)	771.5	281.3	8.6	12.7	19.2	red
6	PI(32:1)	807.5	255.3	118.8	230.9	517.4	grey
7	PI(34:0)	837.5	255.3	83.8	124.0	164.1	red
8	PI(34:1)	835.5	255.3	974.2	1333.1	1817.4	red
9	PI(34:2)	833.5	255.3	1509.0	2022.5	2664.8	red
10	PI(35:2)	847.5	269.3	57.4	79.4	110.0	grey
11	PI(36:1)	863.5	283.3	1303.0	1826.4	2368.1	red
12	PI(36:2)	861.5	281.3<br>283.3	5208.8	6973.8	8807.1	grey
13	PI(36:3)	859.5	283.3<br>281.3<br>255.3	848.0	1185.5	1652.3	grey
14	PI(36:4)	857.5	255.3	997.1	1374.7	1936.5	pink
15	PI(37:4)	871.5	269.3	71.5	103.7	144.7	grey
16	PI(38:2)	889.6	283.3	114.2	177.5	271.3	grey
17	PI(38:3)	887.5	283.3	1264.3	1761.4	2332.6	red
18	PI(38:4)	885.5	283.3<br>281.3	10467.3	14232.2	17750.6	red
19	PI(38:5)	883.5	283.3	155.7	227.3	349.8	grey
20	PI(38:6)	881.5	255.3<br>281.3	143.9	216.0	307.6	pink
21	PI(40:4)	913.6	311.3<br>283.3	111.5	157.2	212.7	red
22	PI(40:5)	911.5	283.3	211.0	306.6	427.3	pink
23	PI(40:6)	909.5	283.3	284.5	403.8	568.2	pink
24	PE(34:1)	718.5	577.5	534.5	784.7	1167.4	red
25	PE(34:2)	716.5	575.5	1311.4	1923.9	2727.5	red
26	PE(34:3)	714.5	573.5	55.4	85.3	139.6	red
27	PE(36:1)	746.5	605.5	1261.1	1772.6	2506.1	red
28	PE(36:2)	744.5	603.5	5150.1	7205.5	10640.1	red
29	PE(36:3)	742.5	601.5	1149.2	1653.9	2419.0	red
30	PE(36:4)	740.5	599.5	1281.2	1788.9	2489.4	red
31	PE(38:3)	770.5	629.5	509.6	708.0	955.4	red
32	PE(38:4)	768.5	627.5	4477.9	6360.2	8657.5	red
33	PE(38:5)	766.5	625.5	1302.5	1901.8	2584.9	red
34	PE(38:6)	764.5	623.5	2057.1	3320.7	4786.8	red
35	PE(38:7)	762.5	621.5	43.3	65.1	90.7	red
36	PE(40:6)	792.5	651.5	1566.6	2533.5	3714.5	red

37	PE(40:8)	788.5	647.5	46.7	67.7	91.6	red
38	PE(O-34:1)	704.5	563.5	87.9	122.1	160.5	black
39	PE(O-34:2)	702.5	561.5	69.8	103.6	145.3	black
40	PE(O-36:2)	730.6	589.6	140.9	228.6	308.6	black
41	PE(O-36:3)	728.5	587.5	90.2	122.2	170.6	black
42	PE(O-36:4)	726.5	585.5	308.6	498.3	752.8	black
43	PE(O-38:2)	758.6	617.6	39.5	52.8	67.7	red
44	PE(O-38:3)	756.6	615.6	24.4	35.4	49.0	black
45	PE(O-38:4)	754.6	613.6	344.7	551.8	775.7	black
46	PE(O-38:5)	752.5	611.5	136.8	206.1	286.3	black
47	PE(O-40:4)	782.6	641.6	74.0	109.8	145.8	black
48	PE(O-40:5)	780.6	639.6	98.3	147.0	220.0	black
49	PE(P-34:1)	702.5	561.5	20.3	36.9	51.8	brown
50	PE(P-34:2)	700.5	559.5	48.9	88.8	130.8	brown
51	PE(P-36:1)	730.6	589.6	27.1	47.3	66.5	brown
52	PE(P-36:2)	728.5	587.5	153.1	253.6	356.1	brown
53	PE(P-36:3)	726.5	585.5	12.8	22.4	35.4	brown
54	PE(P-36:4)	724.5	583.5	130.7	238.4	365.3	brown
55	PE(P-36:5)	722.5	581.5	7.3	13.4	26.4	brown
56	PE(P-38:4)	752.5	611.5	264.0	506.7	718.4	brown
57	PE(P-38:5)	750.5	609.5	165.6	327.5	491.4	brown
58	PE(P-38:6)	748.5	607.5	101.2	202.7	330.3	brown
59	PE(P-40:4)	780.6	639.6	25.3	51.2	74.7	brown
60	PE(P-40:5)	778.6	637.6	28.8	58.6	92.3	brown
61	PE(P-40:6)	776.5	635.5	127.9	262.0	396.7	brown
62	PE(P-40:7)	774.5	633.5	74.1	156.0	235.8	brown
63	PE(P-40:8)	772.5	631.5	10.0	21.4	34.5	brown
64	PE(P-42:4)	808.6	667.6	8.1	12.7	18.2	brown
65	PE(P-42:5)	806.6	665.6	9.5	18.1	27.4	magenta
66	PE(P-42:6)	804.6	663.6	16.0	31.0	49.5	brown
67	PE(P-42:7)	802.6	661.6	7.1	13.7	21.9	brown
68	PE(P-42:8)	800.5	659.5	4.3	8.9	14.2	brown
69	PE(P-44:4)	836.6	695.6	2.2	3.5	5.1	magenta
70	PE(P-44:5)	834.6	693.6	5.9	9.8	14.4	magenta
71	PE(P-44:6)	832.6	691.6	6.3	10.2	15.3	magenta
72	PE(P-44:7)	830.6	689.6	3.9	7.2	11.1	magenta
73	PE(P-44:8)	828.6	687.6	2.5	4.6	7.4	magenta
74	PE(P-46:6)	860.6	719.6	1.0	1.7	2.8	magenta
75	PE(P-46:7)	858.6	717.6	2.4	4.0	6.4	magenta
76	PE(P-46:8)	856.6	715.6	1.6	2.7	4.3	magenta
77	PC(30:0)	706.5	184.1	668.8	1678.0	2949.9	grey
78	PC(32:0)	734.5	184.1	9981.9	12065.0	14318.7	red
79	PC(32:1)	732.5	184.1	1046.0	1535.3	2196.1	red
80	PC(32:2)	730.5	184.1	1361.6	1910.0	2491.6	red
81	PC(34:1)	760.6	184.1	202354.6	256780.0	313300.3	red
82	PC(34:2)	758.5	184.1	411734.0	472010.0	537916.1	grey
83	PC(34:3)	756.5	184.1	12120.2	16194.5	21474.5	red
84	PC(35:1)	774.6	184.1	514.6	676.8	867.0	yellow
85	PC(35:2)	772.6	184.1	5558.2	6941.7	8251.6	yellow

86	PC(35:3)	770.5	184.1	2447.2	3284.5	4199.7	yellow
87	PC(36:1)	788.6	184.1	9172.5	11469.3	14121.1	red
88	PC(36:2)	786.6	184.1	152280.3	184331.9	216448.1	grey
89	PC(36:3)	784.6	184.1	59599.9	73530.4	88964.0	red
90	PC(36:4)	782.5	184.1	76590.1	96994.6	120773.4	pink
91	PC(36:5)	780.5	184.1	4304.4	6736.3	11376.5	grey
92	PC(37:2)	800.6	184.1	1167.0	1448.0	1757.5	yellow
93	PC(37:3)	798.6	184.1	1343.8	1673.6	2033.3	yellow
94	PC(37:4)	796.6	184.1	6977.1	9147.1	11045.7	yellow
95	PC(38:2)	814.6	184.1	5223.8	6669.7	8509.2	red
96	PC(38:3)	812.6	184.1	5861.8	7640.8	9647.3	pink
97	PC(38:4)	810.6	184.1	49489.7	64999.2	80486.9	pink
98	PC(38:5)	808.6	184.1	6668.4	8767.2	11659.7	grey
99	PC(38:6)	806.5	184.1	29599.4	40459.0	54122.8	pink
100	PC(40:4)	838.6	184.1	1404.0	1857.9	2534.2	grey
101	PC(40:5)	836.6	184.1	4068.7	5370.9	6827.8	pink
102	PC(40:6)	834.6	184.1	15099.5	20764.8	27646.1	pink
103	PC(40:7)	832.6	184.1	5675.8	8630.1	11627.3	salmon
104	PC(40:8)	830.5	184.1	776.2	1149.5	1627.0	salmon
105	PC(42:4)	850.6	184.1	374.4	514.4	713.4	grey
106	PC(42:5)	864.6	184.1	474.5	701.3	1089.0	cyan
107	PC(42:6)	862.6	184.1	674.8	1015.3	1483.6	salmon
108	PC(42:7)	860.6	184.1	835.5	1192.2	1787.4	salmon
109	PC(42:8)	858.6	184.1	768.0	1089.5	1478.8	salmon
110	PC(42:9)	856.6	184.1	356.3	521.2	789.6	salmon
111	PC(44:4)	894.7	184.1	204.9	288.2	406.6	grey
112	PC(34:1(OH))	776.6	184.1	75.1	154.0	381.1	grey
113	PC(34:2(OH))	774.5	184.1	213.5	609.9	1924.0	grey
114	PC(36:4(OH))	798.5	184.1	170.7	469.3	2439.5	grey
115	PC(O-32:0)	720.6	184.1	4920.2	6275.9	7825.9	yellow
116	PC(P-34:1)	744.6	184.1	2372.7	3619.8	5032.2	purple
117	PC(P-34:2)	742.6	184.1	6905.4	10278.4	13679.7	purple
118	PC(P-36:2)	770.6	184.1	1168.2	1722.1	2708.6	purple
119	PC(P-36:3)	768.6	184.1	1085.4	1682.5	2346.4	tan
120	PC(P-36:4)	766.6	184.1	71.1	109.4	192.1	tan
121	PC(P-38:4)	794.6	184.1	2822.6	4590.4	6775.4	purple
122	PC(P-38:5)	792.6	184.1	2853.6	4679.0	6872.4	purple
123	PC(P-38:6)	790.5	184.1	1568.7	2509.4	3900.2	purple
124	PC(P-40:4)	822.6	184.1	226.3	350.2	506.5	tan
125	PC(P-40:5)	820.6	184.1	523.8	870.4	1227.5	tan
126	PC(P-40:6)	818.6	184.1	1165.7	1872.1	2814.1	purple
127	PC(P-40:7)	816.6	184.1	618.5	999.2	1499.0	purple
128	PC(P-42:4)	866.5	184.1	256.4	363.2	503.9	tan
129	PC(P-42:5)	848.6	184.1	603.8	886.7	1259.1	greenyellow
130	PC(P-42:6)	846.5	184.1	431.1	661.5	935.6	greenyellow
131	PC(P-44:5)	876.7	184.1	516.7	753.6	1091.0	greenyellow
132	PC(P-44:6)	874.6	184.1	514.5	779.0	1079.5	greenyellow
133	PC(P-44:7)	872.6	184.1	173.0	263.2	369.8	greenyellow
134	PC(P-46:5)	904.7	184.1	336.9	803.2	1323.5	grey

135	PC(P-46:6)	902.7	184.1	41.7	62.9	96.2	tan
136	PC(P-46:7)	900.7	184.1	88.4	135.9	204.0	greenyellow
137	LPE(14:0)	426.2	285.2	0.6	1.2	2.1	red
138	LPE(16:0)	454.3	313.3	155.4	207.2	271.0	blue
139	LPE(18:0)	482.3	341.3	667.3	864.7	1127.1	blue
140	LPE(18:1)	480.3	339.3	347.7	460.8	645.6	red
141	LPE(18:2)	478.3	337.3	276.5	445.8	693.4	grey
142	LPE(20:4)	502.3	361.3	328.3	498.4	806.3	grey
143	LPE(22:4)	530.3	389.3	15.8	28.8	51.6	grey
144	LPE(22:5)	528.3	387.3	31.1	47.5	71.1	grey
145	LPE(22:6)	526.3	385.3	306.6	429.9	638.6	grey
146	LPE(24:0)	566.4	425.4	8.0	10.7	14.1	grey
147	LPE(P-18:0)	466.3	325.3	0.2	0.5	1.2	grey
148	LPC(14:0)	468.3	184.1	377.4	540.8	786.9	blue
149	LPC(16:0)	496.3	184.1	50108.5	61730.6	78434.0	blue
150	LPC(16:1)	494.3	184.1	873.0	1116.6	1451.8	blue
151	LPC(17:0)	510.3	184.1	699.2	954.8	1366.7	blue
152	LPC(18:0)	524.3	184.1	22830.1	28293.6	36269.9	blue
153	LPC(18:1)	522.3	184.1	12225.8	15952.0	20804.7	blue
154	LPC(18:2)	520.3	184.1	19418.6	24593.1	31183.1	blue
155	LPC(18:3)	518.3	184.1	218.5	338.1	510.6	grey
156	LPC(19:0)	538.4	184.1	130.7	185.1	254.9	blue
157	LPC(20:0)	580.4	184.1	199.1	277.9	402.6	blue
158	LPC(20:1)	550.4	184.1	276.1	422.2	669.1	blue
159	LPC(20:2)	548.3	184.1	462.9	606.9	809.1	blue
160	LPC(20:3)	546.3	184.1	1696.1	2292.2	2965.9	blue
161	LPC(20:4)	544.3	184.1	5676.2	7465.4	9520.5	blue
162	LPC(20:5)	542.3	184.1	285.8	480.5	758.6	grey
163	LPC(22:0)	552.4	184.1	32.9	52.2	79.7	grey
164	LPC(22:4)	572.3	184.1	118.6	165.7	240.7	blue
165	LPC(22:5)	570.3	184.1	86.2	128.4	193.5	blue
166	LPC(22:6)	568.3	184.1	1100.6	1610.4	2429.4	blue
167	LPC(24:0)	608.4	184.1	84.6	115.2	159.1	blue
168	LPC(P-16:0)	480.3	184.1	23.7	72.4	118.3	blue
169	LPC(P-18:0)	508.4	184.1	79.8	108.9	149.6	blue
170	SM(d32:1)	675.5	184.1	2978.7	3759.9	4633.9	green
171	SM(d32:2)	673.5	184.1	227.1	293.4	387.5	yellow
172	SM(d33:1)	689.5	184.1	1089.5	1336.6	1613.5	yellow
173	SM(d34:0)	705.6	184.1	2741.8	3143.7	3581.3	green
174	SM(d34:1)	703.6	184.1	46981.8	55629.3	64896.1	green
175	SM(d34:2)	701.5	184.1	5818.5	7445.1	8889.0	green
176	SM(d35:1)	717.6	184.1	658.8	813.8	994.2	yellow
177	SM(d35:2)	715.6	184.1	97.1	124.9	155.3	yellow
178	SM(d36:1)	731.6	184.1	9775.4	12148.5	14801.3	green
179	SM(d36:2)	729.6	184.1	6681.2	8676.9	10762.6	green
180	SM(d36:3)	727.6	184.1	554.6	692.5	821.7	green
181	SM(d37:1)	745.6	184.1	778.3	1023.1	1325.9	yellow
182	SM(d37:2)	743.6	184.1	188.2	241.8	318.2	yellow
183	SM(d38:0)	761.6	184.1	1092.0	1317.8	1545.4	green

184	SM(d38:1)	759.6	184.1	11963.5	14628.2	17368.4	green
185	SM(d38:2)	757.6	184.1	5241.9	6632.6	8021.2	cyan
186	SM(d38:3)	755.6	184.1	239.2	303.6	370.8	yellow
187	SM(d39:1)	773.6	184.1	2441.0	3126.7	3923.9	yellow
188	SM(d39:2)	771.6	184.1	479.1	629.9	797.8	yellow
189	SM(d40:0)	789.7	184.1	2222.7	2751.3	3297.6	green
190	SM(d40:1)	787.6	184.1	28946.8	34791.8	42734.0	green
191	SM(d40:2)	785.6	184.1	19444.3	23572.9	28792.9	green
192	SM(d40:3)	783.6	184.1	1258.9	1586.1	1972.2	cyan
193	SM(d40:4)	781.6	184.1	220.4	273.4	340.6	grey
194	SM(d41:1)	801.7	184.1	9726.8	12546.3	15729.5	grey
195	SM(d41:2)	799.6	184.1	5172.4	6478.4	8085.6	grey
196	SM(d42:1)	815.7	184.1	23195.8	27974.2	33366.5	green
197	SM(d42:2)	813.7	184.1	49006.0	63147.3	81520.8	cyan
198	SM(d42:3)	811.6	184.1	23443.6	30874.6	38871.3	cyan
199	SM(d42:4)	809.6	184.1	1818.2	2648.7	4718.4	grey
